# Prevalence and Risk Factors of Urinary Incontinence in Frail Elderly Females

**DOI:** 10.1155/2020/2425945

**Published:** 2020-04-27

**Authors:** Walaa W. Aly, Hala S. Sweed, Nora A. Mossad, Mohammad F. Tolba

**Affiliations:** Geriatric and Gerontology Department, Faculty of Medicine, Ain Shams University, Cairo, Egypt

## Abstract

*Background*/*Purpose*. Urinary incontinence (UI) is an important geriatric health problem, and it is linked to frailty syndrome. We had conducted a study to detect the prevalence and risk factors of UI and its effect on quality of life (QOL) among frail elderly females living in Cairo, Egypt. *Methods*. We carried out a cross-sectional study on 130 frail elderly females sixty years and older, attending Ain Shams Geriatrics Hospital, Cairo, Egypt. Each patient gave oral consent and then was subjected to history taking, full clinical examination, diagnosis of frailty (clinical frailty scale), assessment of UI by the Arabic version of International Consultation on Incontinence Questionnaire-Urinary Incontinence Short Form (ICIQ-UI SF), assessment of QOL by using the Arabic version of Incontinence Impact Questionnaire Short Form (IIQ-7 SF), and complete urine analysis. *Results*. The prevalence of UI among the studied population was 80%. Mixed UI was the most prevalent type. UI was significantly associated with older age, functional impairment, multiparity, osteoarthritis, stroke, vaginal prolapse, and laxative use. All IIQ-7 subscales were higher (worse health-related QOL) for women with mixed UI. *Conclusion*. Urinary incontinence is prevalent in frail elderly females. Mixed UI, compared with other types, has a significant negative impact on all domains of quality of life.

## 1. Introduction

Urinary incontinence (UI) is defined by the International Continence Society as any involuntary leakage of urine. It is a common clinical problem, and its incidence increases with age [1]. UI is a common symptom in older people, and some reports suggest that UI is associated with frailty, whereby the ability of the body to cope with stress and physiological functions decreases [2].

Normal ageing is not a cause of UI, although age-related changes in lower urinary tract function can predispose older people to UI which is then exacerbated by comorbidities. UI is a major cause of disability and dependency, significantly increasing the risk of care home placement. It also predisposes to career negativity and stress, which itself is a major factor in placement for institutional care [1].

The elderly rank UI among the 4 most distressing disorders after angina, difficulty with ambulation, and psychiatric disorders [3]. UI is frequently associated with a negative impact on quality of life (QOL) of the patient, despite not being a life-threatening condition. UI has many physical and psychological effects on the patients, while at the same time, it is associated with an additional financial burden [4].

Evaluation of the effects of urinary symptoms on the quality of life (QOL) is an important issue. UI may lead to shame, loss of self-esteem, and social isolation [5].

Since incontinence is associated with an increased risk of a global functional impairment, in persons who become incontinent after the age of 65 years [6], this parameter may be an important early marker for signaling the onset of frailty, and the 4^th^ International Consultation on Incontinence has urged researchers to better understand the correlation between UI and frailty [7].

Evaluating the risk factors is an important component in the assessment of UI in the older adults. In both men and women, increased age, genetics, obesity, and tobacco use are associated factors [8]. In women, multiparity is also associated with UI [9].

Screening has the potential to identify urinary incontinence in many women who silently experience its adverse effects but may benefit from appropriate evaluation and treatment. Effective screening may lead to earlier treatment, including behavioral, medical, and surgical interventions, depending on the patient's age and the type and severity of symptoms [10]. Thus a cross-sectional study was conducted to find the prevalence and risk factors of UI and its effect on QOL among frail elderly females living in Cairo, Egypt.

## 2. Materials and Methods

### 2.1. Study Design and Participants

This was a cross-sectional study. The study was carried out at Ain Shams Geriatrics Hospital in Cairo, Egypt.

The study was approved by the ethical committee of Ain Shams University.

The study sample comprised 130 frail females aged sixty years and above.

Exclusion criteria of patients are as follows:

1. Patients who were unwilling to participate
2. Patients with moderate or severe dementia
3. Catheterized patients

### 2.2. General Data Assessment

Each patient gave oral consent and then underwent the following: careful history taking including personal history, age, educational level, marital status, smoking status (current or past), thorough medical history, detailed inquiry about current symptoms, UI risk factors, and medication history. Full clinical examination was done, including calculation of the body mass index (BMI).

### 2.3. Geriatric Domains Assessment

All participants were screened for dementia using the Arabic version by El-Okl et al., [11] of minimental state examination (MMSE) [12] and depression using the Arabic version by Shehata et al. [13] of geriatric depression scale (GDS) [14].

Functional assessment was done using activities of daily living (ADL) [15] and instrumental activities of daily living (IADL) scales [16].

Frailty was assessed by the clinical frailty scale (CFS). The clinical frailty scale is a reliable tool to identify frailty in clinical settings. The CFS ranges from 1 (very fit) to 9 (terminally ill) based on descriptors and pictographs of activity and functional status. According to CFS, study participants were divided into three categories: mildly frail (CFS 5), moderately frail (CFS 6), and severely frail (CFS 7) [17]. Nonfrail subjects (CFS 1-4) were excluded from our study. Of note, we did not include subjects approaching the end of life (CSF 8-9).

Assessment of urinary incontinence was done using the Arabic version of International Consultation on Incontinence Questionnaire-Urinary Incontinence Short Form (ICIQ-UI SF) [18]. The ICIQ-UI SF comprises three-scored items to assess the frequency of urinary incontinence (scores 0–5), the amount of urinary incontinence (scores 0–6), and its impact on the individual's quality of life (scores 0–10). The total score is obtained by adding the scores from these three items together. The higher the score the greater the severity: mild (1–5), moderate (6–12), severe (13–18), and very severe (19–21). There is an unscored self-diagnostic item to assess the perceived causes of leakage.

Assessment of QOL was done using the Arabic version of Incontinence Impact Questionnaire, short form (IIQ-7 SF) [19]. The IIQ-7 SF questionnaire is a seven-item questionnaire designed to assess different domains of QOL impairment. It has a four-point rating scale: 0 = never, 1 = mild, 2 = moderate, and 3 = severe; the higher the score, the poorer the QOL.

Laboratory testing included complete urine analysis. Pyuria was defined as the presence of at least 10 white blood cells per cubic millimeter of centrifuged urine by high power field [20].

### 2.4. Statistical Analysis

The collected data were coded, tabulated, and statistically analyzed using IBM SPSS statistics (Statistical Package for Social Sciences) software version 22.0, IBM Corp., Chicago, USA, 2013.

Description of all data was done in the form of mean (*M*) and standard deviation (SD) for all quantitative variables. Frequency and percentage were used for all qualitative variables. Comparison between quantitative variables was done using *t*-test. Comparison of qualitative variables was done using the chi-square test. Correlations (*r*-value) were assessed by Spearman rank correlation to find relation between different variables. While positive *r*-value indicates direct correlation, negative *r*-value indicates inverse relationship between the variables. The one-way ANOVA test was used to compare between means of more than 2 study groups using the *F* value. *P* value < 0.05 was considered as statistically significant.

## 3. Results

The subjects surveyed in our study were 300 elderly females aged sixty years and above who attended Ain Shams Geriatrics Hospital, Cairo, Egypt, during the period from June 2018 till April 2019. Among them, we included 130 frail elderly females in our study which were selected according to the clinical frailty scale (CFS). The mean age of the frail elderly females was 70.7 ± 8.3 years. Among the 130 frail participants, we found 104 (80%) patients suffering from UI. So we actually included 130 frail participants (who met the frailty criteria), but calculations specific for incontinent subjects (e.g., relationship between type of UI and quality of life) included only 104 patients (i.e., those having any type of UI). This is given in Tables 1, 2, and 3, in which data of incontinent patients were analyzed. In contrast, Tables 4, 5, and 6 (comparing incontinent and continent subjects) included the whole 130 study participants. The prevalence of UI among the studied population was 80%; with a mean duration of 5.9 ± 2.6 years. Mixed UI was the most prevalent type (91%) among participants with UI.

There was a significant relationship between the ICIQ-UI score and the type of UI, being most severe in those with mixed UI (Table 1). There was a statistically significant relationship between ICIQ-UI and the duration of UI as there was a statistically significant higher mean duration of UI among patients with severe and very severe UI in comparison to moderate one according to the ICIQ-UI severity criteria (Tables 2).

As regards to quality of life (QOL) of affected participants using IIQ-7, there was a significant affection of all quality of life domains in patients with mixed UI, compared with other types of UI (Table 3). There was a significant relationship between UI and the presence of depression (positive screening by GDS) (Table 4).

There was a highly significant relationship between UI and frailty (as detected by clinical frailty scale); all moderately and severely frail participants had UI (Table 5). There was a highly significant relationship between frailty category and ICIQ-UI score, being more severe in those participants with more advanced frailty (Table 6).

As regards demographic characteristics and risk factors, participants with UI, compared to those without, were significantly older, more commonly multiparas and had higher degree of functional impairment in both ADL and IADL (Table 7 and 8). As regards associated comorbidities, osteoarthritis, stroke, and vaginal prolapse were significantly more prevalent in participants with UI, compared to those without UI. As regards medication use, laxative use was significantly more prevalent in participants with UI, compared to those without UI.

## 4. Discussion

The study revealed that the prevalence of UI among the studied population was 80%. We found a highly significant relationship between UI and frailty. All moderately/severely frail participants in our study had some type of UI (*P* value < 0.001). In the same way, this study revealed a highly significant relationship between frailty category and severity of UI (ICIQ-UI score), being more severe in those participants with more advanced frailty (*P* value > 0.001).

In agreement with these results, a cross-sectional study done by Wang et al. [21] showed that frailty was more common among subjects with UI than those without UI (60.7% vs. 32.3%, *P* value < 0.001). Chong et al. [2] showed that among 210 participants (mean age 89.4 ± 4.6 years, 69.5% female, 50.0% frail), UI was present in 47.6%, with a higher prevalence among frail individuals (64.8% vs. 30.5%, *P* < 0.001). Incident UI was more common in frail participants (at discharge: 24.3% vs. 9.6%, *P* = 0.038; 6 months: 43.2% vs. 21.7%, *P*=0.020; and 12 months: 56.8% vs. 33.3%, *P*=0.020).

In the study of Kang and Kim [22], participants (*n* = 404) who visited a geriatric clinic were divided into two groups according to the presence or absence of UI (based on questionnaire results) and the relationship between each factor associated with physical frailty and the risk of UI was analyzed. The participants in the UI group showed a weaker grip strength and slower walking speed (*P* < 0.01 and *P*=0.01, respectively) and had more experiences of unintentional weight loss and falls (*P*=0.04 and *P* < 0.01, respectively).

Previous studies reported different prevalence rates of UI among elderly people. This heterogeneity can be explained by differences in participants' characteristics (e.g., sex, mean age, frailty status, and comorbidities), study setting (community, care homes, etc.), and methodological differences.

For example, it was reported that 15–30% of community-dwelling older people have UI. Many studies reported a higher prevalence of UI among care home residents, which ranged between 50% and 80% because UI is associated with older age, frailty, cognitive impairment, and limited mobility leading to a greater level of dependency [1].

As regards type of UI, mixed UI was the most prevalent type of UI among the studied population (91.35% of participants with UI). This large percentage was expected in this frail cohort suffering from functional impairment and multiple comorbidities, making them more likely to have more than one type of UI.

This agree with the study of Talley et al. [23] that showed the most common type of UI in frail women was mixed stress and urgency (62%), followed by urgency (22%), stress (14%), and functional (2%).

Wang et al. [21] conducted a cross-sectional study on 440 participants and found that prevalence of UI was 19.1%. 51.2% of the incontinent subjects had urge incontinence, and 41.7% had functional incontinence.

The current study showed a significant relationship between type of UI and severity of UI (as determined by the ICIQ-UI score), being most severe in those with mixed UI. In agreement with current results, Schreiber Pedersen et al. [24] conducted a postal survey in two regions in Germany and Denmark, including 8000 adult women, and found that women with mixed UI reported a higher mean total ICIQ score in all age groups, than did women with urge UI or stress UI.

There was also a significant relationship between the duration of UI and ICIQ-UI score, being most severe in those with longer duration of UI.

The current study reveals that Incontinence Impact Questionnaire (IIQ-7) scores were higher for women with mixed UI. A significant affection of all quality of life domains was found in participants with mixed urinary incontinence, compared with those suffering from other types of UI. This agrees with the study of Frick et al. [25] who found that IIQ scores were higher for women with mixed incontinence versus urge or stress incontinence (*P* value < 0.01). Yu et al. [26] conducted a cross-sectional study to investigate the impact of UI on QOL using IIQ-7. Their study involved 1608 adult Taiwanese females. They found that women with mixed UI had a higher IIQ-7 score compared to those with stress or urge UI.

This study revealed a significant affection of praying in patients with mixed UI, compared with other types of UI. Yet this result could not be compared to studies done in different non-Muslim populations because praying is actually not an item in the original IIQ and was added in the Arabic version to suit our population. The affection is expected to be influenced most by the amount and the frequency of urine loss. Studies in Muslim women revealed that UI led to the limitation of religious life, related to the need of cleanliness for the practice of prayer [27].

The current study also showed a significant affection of physical activities in patients with mixed UI, compared with other types of UI. In a similar way, Manonai et al. [28] found that mixed UI has the greater impact on physical activities (20.4%) than urge UI (11.5%) and stress UI (9.1%) in their cross-sectional study that involved 319 females with UI. They measured the impact of UI on QOL using IIQ-7.

As regards affection of social life, we found a significant affection of social activities in patients with mixed UI, compared with other types of UI. Manonai et al. [28] reported similar results in their cross-sectional study. They found that mixed UI has the greatest impact on social life (15.6%) followed by urge UI (10.3%) and stress UI (5.5%), respectively.

Moreover, the current study revealed marked affection of travelling in patients with mixed UI, compared with other types of UI. In agreement with this result, Manonai et al. [28] found that mixed UI has the greatest impact on travelling (25.3%) followed by urge UI (19.2%), with the lowest affection found in stress UI (7.3%).

The current study showed a significant relationship between UI and the presence of depressive symptoms (positive screening by geriatric depression scale, GDS) where 89% of depressed participants had UI (*P* value = 0.026). No significant relationship was found between the type of UI and the presence of depressive symptoms. One of the comorbidities associated with UI include depression [29].

As regards emotional wellbeing, significant affection (in the form of depression/hopelessness and anxiety/frustration) was found in patients with mixed UI, compared with other types of UI. This agrees with the results of the study of Manonai et al. [28]. The authors found that mixed UI has the greatest impact on emotional life (30.6%) followed by urge UI (23.1%) and the least affection was found in stress UI (16.4%). This also agrees with the cross-sectional study done by Wang et al. [21], in which the authors concluded that subjects with UI had more depressive symptoms (GDS-5, *P* value = 0.02).

Many cross-sectional studies reported the association between UI and depression. These include a large US population-based cross-sectional study [29] and another smaller Japanese study [30].

In contrast, negative findings were reported in a Korean study by Song and Bae [31]. Another study by Ko et al. [32] did not found an association between UI and self-reported sadness. These negative findings may reflect differences in definition of depression and patient selection criteria.

UI has many risk factors, which we inquired about in our study. For instance, there was a significant relationship between parity and the presence of UI. Most of the study participants with UI were multiparas (78.85%, *P* value = 0.005). An epidemiological study in Italy showed that vaginal birth increased the risk of stress UI (OR 3.8 vs. nulliparous) but not of urge UI [33]. Fabio et al. [34] confirmed these findings with regard to reproduction; history of vaginal births increased the risk of stress and mixed UI but not of urge UI and overactive bladder.

As regards obesity, the current study failed to prove the relation between UI and obesity. In contrast, Fabio et al. [34] found that the risk of all types of UI is increased in women with high body mass index. This contradiction may be explained by the fact that our study included less number of obese subjects (38 participants, 29%). Of note, 28 of them had UI, while 10 only did not suffer from UI, but this difference did not reach statistical significance.

The relationship between smoking and UI is unclear. Heavy current/former smokers have been shown to have a higher risk of UI [35]. Our data and that of others (Fabio et al. [34]; Burgio et al. [36]), however, have failed to confirm this relationship.

It is worthwhile mentioning that current study included a few number of smokers (16.92% of the whole study sample) because our population consisted of female participants only. Among 22 smokers included in our study, 19 had UI.

As regards functional limitation, participants with UI, compared to those without, had higher degree of functional impairment in both activities of daily living (ADL) and instrumental activities of daily living (IADL). Many studies reported a strong association between functional impairment and UI. For instance, a cross-sectional study done by Wang et al. [21] showed that subjects with UI had poorer physical function.

It is important to consider a patient's drug history, particularly in new-onset incontinence and in the elderly, where polypharmacy is common [37]. Wang et al. [21] showed that subjects with UI had more polypharmacy.

Drugs that are commonly associated with UI include sedative-hypnotics, diuretics, anticholinergics, antispasmodics, analgesics, antihistamines, antipsychotics, alpha-adrenergic agonists, alpha-antagonists, calcium-channel blockers, angiotensin-converting enzyme inhibitors (ACEIs), and antiparkinsonian medications [38–40].

The current study showed a significant relationship between UI and laxative use (*P* value = 0.043). Similar result was reported by Blekken et al. [41] who studied 261 patients and concluded that UI was associated with constipation and laxative use.

Comorbidities are common in the older adult population, and UI can be caused by, associated with, or worsened by these. One study found UI to be independently associated with having at least one geriatric condition in 60% of study participants, at least two in 29% and at least three in 13% [42]. In the current study, UI was significantly associated with osteoarthritis, stroke, and vaginal prolapse.

This agrees with Turner-Stokes and Frank [43] proved that arthritis is associated with impaired mobility and dexterity and can lead to functional UI. Shaw and Wagg [42] concluded that UI is commonly associated with neurological conditions, including stroke, dementia, and Parkinson's disease. In the same way, Pandeva et al. [44] reported on 152 female participants in a tertiary referral center. The pelvic organ prolapse symptom of a vaginal bulge was present in 11% (17/152). Among cases of vaginal prolapse, 59% were accompanied by UI.

## 5. Conclusion

Urinary incontinence is prevalent in frail elderly females. Quality of life is significantly impaired in patients with UI, especially with mixed type and longer duration of symptoms. UI is significantly associated with older age, functional impairment, multiparity, osteoarthritis, stroke, vaginal prolapse, and laxative use.

### 5.1. Strengths of This Study

This study has several strengths. First, it confirmed a high prevalence of UI (an important geriatric syndrome) with the application of multiple assessment tools in Arabic language in frail elderly females, a population relatively less included in previous research studies in our country. Second, we analyzed a wide range of risk factors/comorbidities in relation to UI in frail elderly females. Third, the study findings add to the growing body of literature on predictors and risk factors of UI in frail elderly Egyptian subjects.

### 5.2. Limitations of This Study

Our study has some limitations. The relatively small sample sizes make generalizability of study findings cautious. The use of a cross-sectional design did not allow comparison with nonfrail subjects.

## Figures and Tables

**Table 1:** Relationship between types of urinary incontinence and ICIQ-UI (severity of urinary incontinence).

UI type	ICIQ-UI								Chi-square	
	Moderate		Severe		Very severe		Total			
	*N*	%	*N*	%	*N*	%	*N*	%	*X* ^2^	*P* value
Urge	1	2.44	0	0.00	0	0.00	1	0.96	13.238	0.039^*∗*^
Stress	5	12.20	0	0.00	0	0.00	5	4.81		
Functional	2	4.88	1	1.82	0	0.00	3	2.88		
Mixed	33	80.49	54	98.18	8	100.00	95	91.35		
Total	41	100.00	55	100.00	8	100.00	104	100.00		

ICIQ-UI: International Consultation on Incontinence Questionnaire-Urinary Incontinence; UI: urinary incontinence.

**Table 2:** Relationship between duration of urinary incontinence (in all patients with UI) and ICIQ-UI (severity of urinary incontinence).

ICIQ total	*N*	UI duration (years)		ANOVA	
		Range	Mean ± SD	*F*	*P* value
Moderate	41	2–15	4.780 ± 2.752	8.014	0.001^*∗*^
Severe	55	2–10	6.600 ± 2.051		
Very severe	8	2–10	7.375 ± 3.204		

ICIQ: International Consultation on Incontinence Questionnaire; UI: urinary incontinence.

**Table 3:** Relationship between types of urinary incontinence and quality of life (QOL).

QOL domain	UI type	IIQ-7								Chi-square	
		Never		Mild		Moderate		Severe			
		*N*	%	*N*	%	*N*	%	*N*	%	*X* ^2^	*P* value
Praying	Urge	0	0.00	1	3.45	0	0.00	0	0.00	45.656	<0.001^*∗*^
	Stress	0	0.00	4	13.79	0	0.00	1	4.76		
	Functional	1	100.00	0	0.00	2	3.77	0	0.00		
	Mixed	0	0.00	24	82.76	51	96.23	20	95.24		

House keeping	Urge	0	0.00	1	3.45	0	0.00	0	0.00	23.732	0.005^*∗*^
	Stress	2	40.00	1	3.45	2	3.45	0	0.00		
	Functional	1	20.00	1	3.45	1	1.72	0	0.00		
	Mixed	2	40.00	26	89.66	55	94.83	12	100.00		

Physical recreational activities	Urge	0	0.00	1	4.17	0	0.00	0	0.00	26.024	0.002^*∗*^
	Stress	2	40.00	1	4.17	2	3.77	0	0.00		
	Functional	1	20.00	1	4.17	0	0.00	1	4.55		
	Mixed	2	40.00	21	87.50	51	96.23	21	95.45		

Social activities	Urge	0	0.00	1	4.35	0	0.00	0	0.00	33.092	<0.001^*∗*^
	Stress	2	50.00	2	8.70	1	2.22	0	0.00		
	Functional	1	25.00	0	0.00	1	2.22	1	3.13		
	Mixed	1	25.00	20	86.96	43	95.56	31	96.88		

Travelling	Urge	0	0.00	0	0.00	1	2.50	0	0.00	24.984	0.003^*∗*^
	Stress	1	33.33	3	15.00	0	0.00	1	2.44		
	Functional	1	33.33	0	0.00	1	2.50	1	2.44		
	Mixed	1	33.33	17	85.00	38	95.00	39	95.12		

Anxiety/frustration	Urge	0	0.00	1	2.78	0	0.00	0	0.00	27.511	0.001^*∗*^
	Stress	1	100.00	3	8.33	1	2.04	0	0.00		
	Functional	0	0.00	2	5.56	0	0.00	1	5.56		
	Mixed	0	0.00	30	83.33	48	97.96	17	94.44		

Depression/hopelessness	Urge	1	33.33	0	0.00	0	0.00	0	0.00	43.339	<0.001^*∗*^
	Stress	1	33.33	3	7.50	1	2.17	0	0.00		
	Functional	0	0.00	2	5.00	1	2.17	0	0.00		
	Mixed	1	33.33	35	87.50	44	95.65	15	100.00		

IIQ-7: Incontinence Impact Questionnaire-7; QOL: quality of life.

**Table 4:** Relationship between urinary incontinence and depression.

UI	GDS				Chi-square	
	Depressed		Not depressed			
	*N*	%	*N*	%	*X* ^2^	*P* value
Yes	49	89.09	55	73.33	4.924	0.026^*∗*^
No	6	10.91	20	26.67		
Total	55	100.00	75	100.00		

GDS: geriatric depression scale; UI: urinary incontinence.

**Table 5:** Relationship between urinary incontinence and clinical frailty scale.

UI	Clinical frailty scale								Chi-square	
	Mildly frail		Moderately frail		Severely frail		Total			
	*N*	%	*N*	%	*N*	%	*N*	%	*X* ^2^	*P* value
Yes	62	70.45	27	100.00	15	100.00	104	80.00	15.511	<0.001^*∗*^
No	26	29.55	0	0.00	0	0.00	26	20.00		
Total	88	100.00	27	100.00	15	100.00	130	100.00		

UI: urinary incontinence.

**Table 6:** Relationship between severity of urinary incontinence and clinical frailty scale.

ICIQ total	Clinical frailty scale								Chi-square	
	Mildly frail		Moderately frail		Severely frail		Total			
	*N*	%	*N*	%	*N*	%	*N*	%	*X* ^2^	*P* value
Moderate	35	56.45	6	22.22	0	0.00	41	39.42	50.763	<0.001^*∗*^
Severe	27	43.55	20	74.07	8	53.33	55	52.88		
Very severe	0	0.00	1	3.70	7	46.67	8	7.69		
Total	62	100.00	27	100.00	15	100.00	104	100.00		

ICIQ-UI: International Consultation on Incontinence Questionnaire-Urinary Incontinence.

**Table 7:** Comparison between participants with and without urinary incontinence as regards age.

Age	UI		*T*-test	
	Yes	No	*T*	*P* value
Range	60–91	60–68	4.966	<0.001^*∗*^
Mean ± SD	72.308 ± 8.357	64.039 ± 2.735		

UI: urinary incontinence.

**Table 8:** Comparison between participants with and without urinary incontinence as regards demographic characteristics and risk factors.

		UI						Chi-square	
		Yes		No		Total			
		*N*	%	*N*	%	*N*	%	*X* ^2^	*P* value
Smoking	Yes	19	18.27	3	11.54	22	16.92	0.670	0.413
	No	85	81.73	23	88.46	108	83.08		

Obesity	Yes	28	26.92	10	38.46	38	29.23	1.339	0.247
	No	76	73.08	16	61.54	92	70.77		

Parity	Nullipara	6	5.77	6	23.08	12	9.23	12.662	0.005^*∗*^
	Multipara	82	78.85	13	50.00	95	73.08		
	CS	7	6.73	5	19.23	12	9.23		
	CS and vaginal	9	8.65	2	7.69	11	8.46		

ADL	Independent	45	43.27	19	73.08	64	49.23	8.371	0.015^*∗*^
	Assisted	36	34.62	6	23.08	42	32.31		
	Dependent	23	22.12	1	3.85	24	18.46		

IADL	Assisted	46	44.23	20	76.92	66	50.77	8.894	0.003^*∗*^
	Dependent	58	55.77	6	23.08	64	49.23		

Pyuria	Yes	48	46.15	14	53.85	62	47.69	0.493	0.482
	No	56	53.85	12	46.15	68	52.31		

Hysterectomy	Yes	21	20.19	2	7.69	23	17.69	2.232	0.135
	No	83	79.81	24	92.31	107	82.31		

Hernia repair	Yes	10	9.62	4	15.38	14	10.77	0.720	0.396
	No	94	90.38	22	84.62	116	89.23		

Spinal surgery	Yes	4	3.85	0	0.00	4	3.08	1.032	0.310
	No	100	96.15	26	100.00	126	96.92		

UI: urinary incontinence; nullipara: females who never gave birth; multipara: females who have two or more pregnancies (all were vaginal delivered); CS: cesarean section; ADL: activities of daily living; IADL: instrumental activities of daily living.

## Data Availability

All the data used to support the findings of this study are included within the article and any further data can be provided from the corresponding author upon reasonable request.
